# Role of the oxide in memristive quasi-1D silicon nanowires†

**DOI:** 10.1039/d5nr00104h

**Published:** 2025-02-19

**Authors:** Junrui Chen, Kapil Bhardwaj, Sandro Carrara

**Affiliations:** a Bio/CMOS Interfaces Lab, Institute of Electrical and Micro Engineering, Engineering Faculty, École Polytechnique Fédérale de Lausanne Rue de la Maladiere 71b Neuchatel 2000 Switzerland junrui.chen@epfl.ch

## Abstract

Memristors are garnering significant attention due to their high similarity to biological neurons and synapses, alongside their unique physical mechanisms. Biosensors exhibiting memristive behaviour have demonstrated substantial efficacy in detecting therapeutic and biological compounds in the past decade. This report investigates silicon nanowire (SiNW)-based devices incorporating Schottky barriers, which exhibit potential for memristive behaviour. The SiNWs are fabricated between two nickel (Ni) pads, defined as 1.5 μm in length and 90 nm in width, then forming a quasi-one-dimensional (1D) back-to-back Schottky diode structure due to their large aspect ratio. After oxygen plasma treatment of the SiNW, this back-to-back diode structure begins to exhibit memristive behaviour. Our experimental data indicate that this behaviour is induced by superficial oxygen along the SiNW and is influenced by the contacts within the Schottky barrier and the intermediate silicon oxide layer. Furthermore, we have developed a mathematical model derived from the thermal emission equation of Schottky diodes to accurately characterize and understand this memristive behaviour. Thanks to this model, it is possible to accurately fine-tune the design of memristive devices for application in neuromorphic computing and memristive biosensing.

## Abstract 2

Memristive behavior exhibits after surface oxidation.

## Graphical abstract

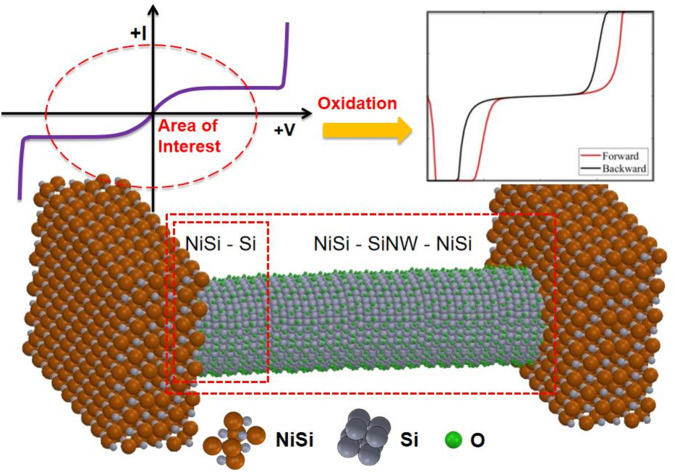

## 1. Introduction

Since the introduction of the solid-state memristive device based on TiO_2_ by Strukov and Williams in 2008,^1^ memristive devices have generated significant attention in various domains of application. This high interest can be attributed to their distinctive nonlinear characteristics and exceptional switching properties, including but not limited to low energy consumption, fast switching speed, and high endurance.^2–5^ Notably, memristive devices have firmly established themselves as a potential component in applications such as memory computing^6^ and neural networks.^7^ Generally speaking, memristors are categorized into non-volatile and volatile switching behaviours.^8^ Non-volatile memristors retain information even after the power is turned off, with physical mechanisms rooted to electron spins, ferroelectric polarization, crystalline–amorphous transitions, or interplay between ions and electrons. On the other hand, volatile memristors exhibit spontaneous conductance decay once electrical or optical stimulation stops.^8^ Compared to non-volatile devices, volatile memristors possess a unique combination of high similarity to biological neurons and synapses, along with distinctive physical mechanisms.^9^

The memristive property was leveraged for the first time in the bio-sensing field by our group in 2011, under the term “memristive biosensor”.^10^ An extensive review detailing several “memristive biosensors” can be found in the study by Carrara.^11^ In the context of biosensing, SiNWs are particularly significant due to their substantial aspect ratio and role as a Bio/CMOS interface. SiNW-based biosensors have demonstrated their efficacy in detecting a range of biomolecules, including DNA, proteins, and therapeutic compounds.^12–14^ A thorough examination of their applications in carcinoma diagnosis is provided in the comprehensive review by M.-A. Doucey and S. Carrara.^15^ SiNW-based memristive biosensors have shown remarkable potential as highly sensitive tools for protein measurement, enabling precise and efficient detection in diverse biological contexts.^16,17^ These advancements highlight the far-reaching implications and multifold nature of memristive device research in academia. A critical observation is that upon biofunctionalization, the characteristic memristive pinched current–voltage (*I*–*V*) curves are typically lost, leading to the definition of a new parameter known as the “voltage gap”,^18^ and it has been observed for the first time in naked nanowires too.^19^ This voltage gap exhibits a proportionality to the concentration of target biomolecules, returning unprecedented limits-of-detection as a biosensing parameter. Collectively, these developments highlight the vast potential and broad applicability of memristive devices in the field of biosensors. In this field, we conclude from our past results that our memristive devices based on SiNWs predominantly exhibit a non-crossing anti-clockwise loop, indicating a volatile resistive switching behaviour. A recent study highlights that the *I*–*V* hysteresis exhibited by bio-functionalized nanowires is determined by their inherent memristive characteristics and the induced capacitive effect caused by the charged biomolecules.^20^ This insight further emphasizes the potential of SiNW-based memristive biosensors in detecting and analyzing biological compounds with high sensitivity and specificity.

Nevertheless, the mechanism responsible for the origin of this volatile memristive behaviour in quasi-1D structure still eludes a full understanding. The resistive switching effect produced by most of the popular memristive architectures belongs to the formation of a conductive filament (CF) inside a dielectric medium.^21–23^ The CF memristive device is typically known for its characteristic “sandwich-like” structure,^24^ which comprises a thin dielectric layer positioned between the bottom and top electrodes, *e.g.*, in a highly limited volume toward the quasi-0 structure. On the other hand, resistive switching has been observed in several kinds of 1D and 2D structures made of, while not limited to, silicon,^19^ gold,^25^ graphene,^26^ and organic polymers.^27^ These further switching structures are challenging since they do not present the possibility of forming conductive filaments, due to the long length of the conductive channel. Therefore, 1D memristive devices are not simply explained by the CF model.

In addition, the utilization of metal–semiconductor (Schottky) contacts has emerged as a promising alternative for non-filamentary memristive devices, as evidenced by several academic studies.^28–34^ Notably, a recent research endeavour led by Zhou *et al.*^35^ introduces a two-dimensional structure based on back-to-back Schottky diodes connected through a planar channel semiconductor. The resulting device exhibits both crossing and non-crossing hysteresis loops for varying voltage sweep, which are associated with volatile and non-volatile switching,^36^ respectively. The switching mechanism in the reported structure has been attributed to the migration of defects, resulting in the redistribution of defect concentration within the device. Consequently, alterations in defect concentration and the Schottky barrier at the contacts lead to variations in contact resistance as well as in the resistance of the semiconductor layer. Thus, Schottky-diode-modulated memristive devices basically rely on the interplay of various mechanisms, including defect redistribution, charge carrier mobility, Schottky barrier height (SBH), and interface properties. Changes in any of these factors can impact the memristive properties of the device.^36^

Interestingly, an effective quasi-1D memristive device has been realized utilizing a ZnO nanowire, incorporating Schottky contacts in both terminals.^37^ In this device, the induction of a low-resistance state is achieved through the accumulation of vacancies in the barrier enhancement layer, resulting in the formation of local conductive filament channels upon the application of external voltage. By tuning the set/reset voltage, the ZnO based device presents non-volatile memristive behaviour. Besides tuning of the SBH, the trap-assisted tunneling effect through the Schottky barrier at the metal electrode contact has been demonstrated as a pivotal factor in eliciting both volatile and non-volatile memristive behaviour, particularly in oxide-based devices.^38^

Enhancing the comprehension of the mechanisms and modelling underlying volatile memristive behaviour is crucial for optimizing the fabrication of memristive biosensors. This is particularly significant when considering the modeling of the capacitive-coupled effect induced by absorbed biomolecules.^20,39^ Therefore, this report aims to utilize experimental data to uncover the origins of volatile memristive behaviour and to identify the factors influencing the switching properties in SiNW-based memristors.

The report describes the fabrication of the devices, realized with central SiNWs connected between two nickel/nickel silicide (Ni/NiSi) electrodes, which form a back-to-back diode structure connected through a silicon channel. Our findings, both experimental and theoretical, clearly demonstrate that a pure diode structure is unable to explain the measured hysteresis. Robust volatile memristive properties are observed after the device undergoes native oxidation or oxygen plasma treatments. The extent of hysteresis is highly influenced by the SBH and by the characteristics of the oxide intermediate layer.

Model simulations perfectly align with the fabricated device and demonstrate no hysteresis for the newly fabricated samples. To explain the influence of the surface oxide layer, an energy-based model is introduced, revealing the origin of the volatile memristive behaviour.

The main scientific contributions of this article are as follows:

1. Developing the robust memristive behaviour in a quasi-1D nanostructure;

2. Experimentally demonstrating that the surface oxide layer is the key factor inducing memristive behaviour, with its properties influenced by Schottky contacts.

3. Promoting an energy-based model that closely aligns with the observed behaviour.

## 2. Experiment and methods

### 2.1. Standard device fabrication

A standard fabrication process for SiNWs began with a top-down, CMOS-compatible technique on a p-type silicon-on-insulator (SOI) substrate with a resistivity range of 14–22 Ω cm. The substrate consisted of a thin silicon layer and a buried oxide layer, supported by a thicker silicon wafer. To define the pattern for the Ni pads, a layer of polymethyl methacrylate (PMMA) was precisely patterned using electron beam lithography (EBL). Subsequently, a 50 nm thick layer of nickel (Ni) was evaporated onto the surface. Then, the excess PMMA layer was removed with acetone, followed by ultrasonic rinsing for 20 minutes. Consequently, the patterned Ni remained on the substrate through the lift-off process. The substrates, carrying the patterned Ni layer, underwent an annealing step in an inert N2 atmosphere under varying annealing conditions. The default annealing conditions for the standard device were at 400 °C for 20 minutes. This annealing process initiated a reaction between Ni and silicon from the substrate, forming Schottky contacts.

Another EBL process was employed using a 100 nm thick layer of hydrogen silsesquioxane (HSQ) as a negative-tone resist. After exposure and development, the substrate underwent deep reactive ion etching (DRIE), also known as the “Bosch process”, to create the silicon nanowires. This process involved alternating etching and passivation steps using SF_6_ (etching gas) and C_4_F_8_ (passivation gas) in a cyclic manner. As a result, stacked silicon nanowires were formed, leaving them suspended between the NiSi contacts. Lastly, the HSQ resist was stripped in a solution comprising 1% HF for 1 minute. The application of 1% HF served a dual purpose: it stripped the HSQ resist and potentially eliminated oxide layers from the SiNW surface. A more comprehensive schematic can be found in Fig. S1.† The defined SiNWs were captured using a scanning electron microscope (SEM).

### 2.2. Device characterization

To characterize the device's behaviour, *I*–*V* characteristics were measured by connecting the device to a probe station using tungsten needles at a constant back-gate potential of 0 V. The probe station interfaced with a Sub-Femtoamp 6340 Remote Source Meter, designed by Keithley. This setup efficiently captured and analysed the intricate *I*–*V* characteristics, providing comprehensive insights into the memristive behaviour.

### 2.3. Surface oxide layer on the silicon nanowires

Since the standard device established with HF solution removed the surface oxide layer, the device showed the behaviour of a pure back-to-back diode structure. The *I*–*V* curve was obtained using the source meter. The voltage window to characterize the memristive behaviour was 4 V, with a default scanning rate of 1 V s^−1^. A series of scanning rates—0.5 V s^−1^, 1 V s^−1^, 2 V s^−1^, and 4 V s^−1^—were employed to analyse the frequency-dependent memristive behaviour. Two parallel strategies were employed to observe the effect of the superficial oxide layer:

#### 2.3.1 Native oxidation

The new device with a pure back-to-back structure was exposed to open air. The *I*–*V* curves were monitored over the course of one week.

#### 2.3.2 Oxygen plasma treatment

After immersing in HF solution, the device was treated with oxygen plasma immediately in the “Tepla GiGA- batch”. The oxygen gas flow rate was maintained at 800 ml min^−1^ and the plasma power at 600 W for 15 min to guarantee sufficient oxidation of the silicon nanowire surface.

### 2.4. Schottky barrier tuning

Varying nickel-to-silicon (Ni : Si) ratios effectively adjusted the SBH at the contacts.^40^ Changes in silicidation temperature and duration had an effect as well. The silicide interface layer gave rise to an increased carrier injection;^41^ hence, a more pronounced melting of Ni and Si atoms led to a reduction in the SBH.^40^ We explored three distinct scenarios for the formation of Schottky contacts. Following nickel evaporation, one of the devices underwent silicidation omission, while the remaining two underwent annealing at a temperature of 400 °C for a duration of 20 minutes or 40 minutes, respectively. The color change of the contacts was observed with microscopy, and the interface conditions were captured using back-scattered electron detection (BSD).

### 2.5. Intermediate oxide layer

Before the definition of the Ni pattern, a thin silicon dioxide layer was sputtered with “Pfeiffer SPIDER 600” onto the top silicon surface, with thicknesses calculated to be 10 nm and 20 nm. After the device was fabricated, an intermediate oxide layer existed between the Ni and Si layers.

## 3. Results

**Fig. 1:**
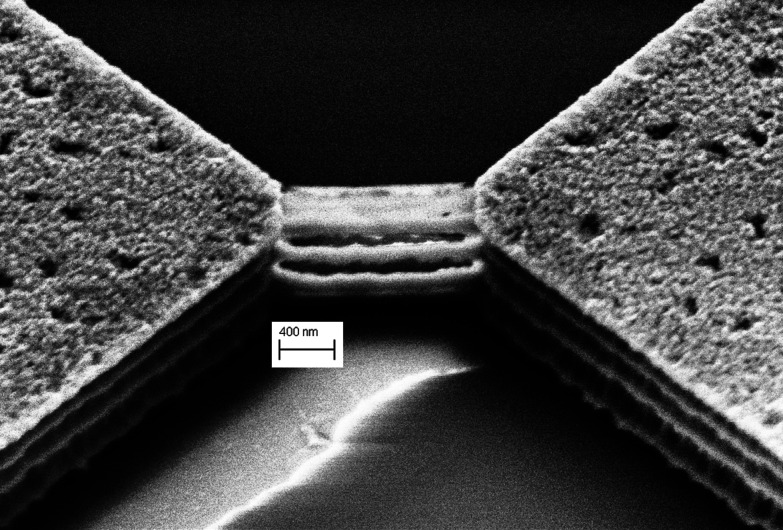
SEM capture of the NiSi-SiNW-NiSi device, tilted 40 degrees.

As captured using the scanning electron microscope (SEM) (Fig. 1), the device features two NiSi pads and four stacked SiNWs positioned in between. The SiNWs are approximately 1.45 μm long and 91 nm wide.

### 3.1. Surface oxide layer on the silicon nanowire

Over the past decade of biosensor research, our group has consistently observed memristive pinched-hysteresis-loop characteristics in the same fabricated devices before biofunctionalization.^5,42–44^ It is even more interesting to note that some previous work can be found which demonstrates that the Schottky diode-based back-to-back structure exhibits various types of memristive properties, relying on diverse mechanisms.^34,36^ In this new paper, we are instead going to elucidate the different effects of the oxide layers as well as the role of the Schottky barrier in the performances of our devices.

#### 3.1.1 Native oxidation

**Fig. 2:**
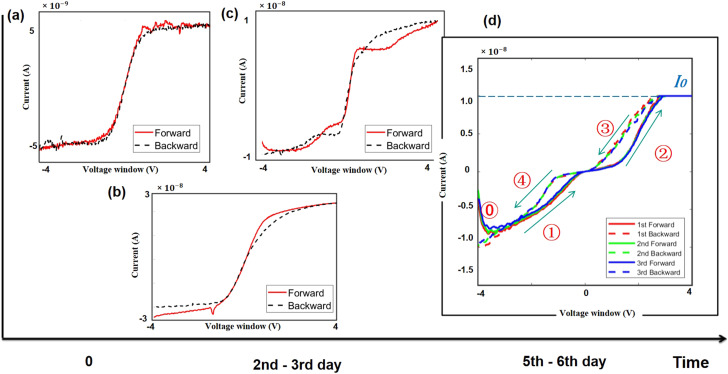
*I*–*V* curve of (a) the brand new fabricated device and (b and c) the device gradually oxidized naturally, showing unpredictable and uncontrollable behaviour. (d) Repeated volatile hysteresis after five days; the oxidation process reaches an equilibrium state.

We have made an interesting observation of our device characteristics over time. Considering the propensity for silicon oxidation in an open environment, Fig. 2 presents the evolving electrical characteristics in the *I*–*V* plane over time.

Fig. 2(a) presents the *I*–*V* curve of the brand new fabricated device. It clearly shows a typical electrical property of a back-to-back diode structure. When an external voltage is applied to the device, there is always one diode that is reverse biased, indicating that the maximum current is constrained by the Schottky contacts as saturation current *I*_0_ described by a typical thermal emission model in eqn (1),^45^1
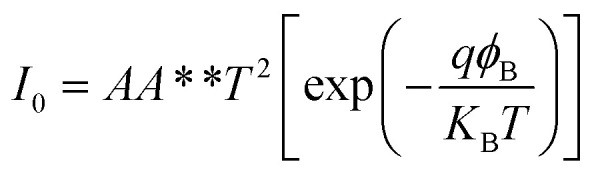
where *k*_B_ is the Boltzmann constant, *T* is the absolute temperature, *A* is the area of the Schottky contact, A** is the effective Richardson constant of the semiconductor, and *ϕ*_B_ is the SBH of a p-type Schottky contact. In the given expression, all parameters, with the exception of the applied voltage, are assumed to remain constant at room temperature. Consequently, during the forward and backward sweep, there is no hysteresis observed, indicating the absence of the memristive behaviour.

With the native oxidation of the silicon nanowires, and then with the oxygen progressively doping the silicon surface, the *I*–*V* curve during this period (Fig. 2(b and c)) manifests an unpredictable resistance state, exhibiting diverse memristive characteristics. As the oxidation process in open air approaches equilibrium after five days, the *I*–*V* curve attains a relatively stable state, revealing pronounced and repeatable analog volatile hysteretic behaviour, as presented in Fig. 2(d). An abrupt resistance change occurs when the sweep starts at −4 V in phase ⓪. As the voltage increases, the current eventually stabilizes at the saturation current *I*_0_, as depicted in Fig. 2(a–c), constrained by the Schottky contacts. Phases ① and ③ exhibit a low resistance state as the voltage decreases, while phases ② and ④ exhibit a high resistance state as the voltage increases. From Fig. 2(d), we can conclude that the resistive-switching occurs before the current reaches its maximum *I*_0_.

#### 3.1.2 Oxygen plasma treatment

To avoid the ambiguous hysteretic characteristics as shown in Fig. 2(b and c), the devices are oxidized under a sufficient oxygen plasma atmosphere at 600 W for 15 minutes. On the other hand, oxygen plasma treatment is also employed to eliminate the organic residuals and activate the silicon surface prior to biofunctionalization.^44^ During the oxygen plasma treatment, the SiNWs undergo substantial ion generation within their bulk region due to the absorbed oxygen atoms, resulting in the modification of the physical properties of the SiNWs.^46^ Thus, in real-time operation, high-power oxygen plasma treatment can enhance the charge-carrier concentration in the nanowires. Considering the quasi-1D nature of the device due to the extensive length of SiNWs, the impact of the oxygen plasma on bulk silicon is considerably more dominant than its effect on the Schottky contacts.

**Fig. 3:**
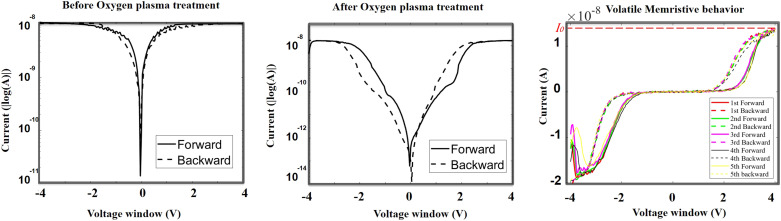
The log (*I*)–*V* curve before (a) and after (b) oxygen plasma treatment. (c) Repeatable and robust volatile memristive behaviour after oxygen plasma treatment in the non-log curve.

As the devices are newly fabricated, the hysteresis is negligible as presented in the semi-logarithmic curve shown in Fig. 3(a). After oxygen plasma treatment, a very evident hysteresis appears, as shown in Fig. 3(b). A repeatable volatile memristive behaviour following oxygen plasma treatment within a 4 V voltage window is depicted in Fig. 3, referring to the demonstration of the stability of the device. Fig. 3(c) shows the same anti-clockwise pinched hysteresis previously obtained with the natural oxidation process illustrated in Fig. 2(d). Consistently with the observations in Fig. 2(d), an abrupt conductance change is evident when the sweep commences at −4 V.

**Fig. 4:**
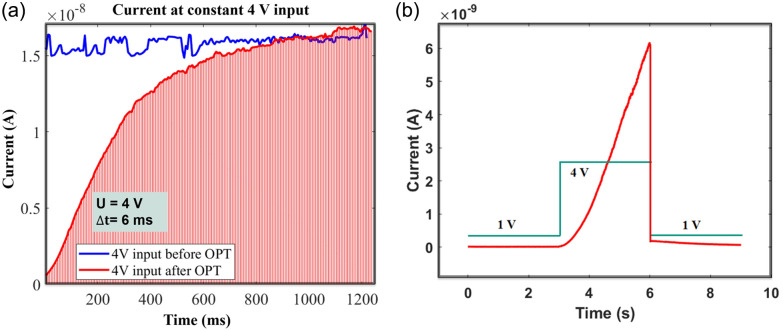
(a) Current measurement at constant 4 V input before and after oxygen plasma treatment (OPT). (b) Volatility test.

The reason for this abrupt conductance change is distinctly elucidated by applying a constant external voltage to the device before and after the oxygen plasma treatment. The voltage remains at 4 V; meanwhile, 200 measurements are acquired each at 6 ms. The resulting *I*–*V* curves are shown in Fig. 4. With the device before the plasma treatment, the current remains around 15 nA, consistent with the constrained current observed in Fig. 2(a). On the other hand, a clear variation in conductance is observed in the device as measured after the oxygen plasma treatment. The current gradually increases from almost 0 A to approximately 12 nA, indicating a transition from a high-resistance state to a low-resistance state. This conductance change corresponds to the phase labeled ⓪ in Fig. 2(d). These data clearly demonstrate that the injection of carriers due to an applied voltage as per the memristive effect is possible only in the presence of oxygen in the silicon nanowires. Moreover, when the input starts at 1 V (Fig. 4b), the current remains around 10 pA. Upon increasing the voltage to 4 V, the current rises gradually, indicating a transition to the switch-on state. When the voltage returns to 1 V, the current decreases accordingly, reverting to the switch-off state, thereby demonstrating volatile behaviour.

**Fig. 5:**
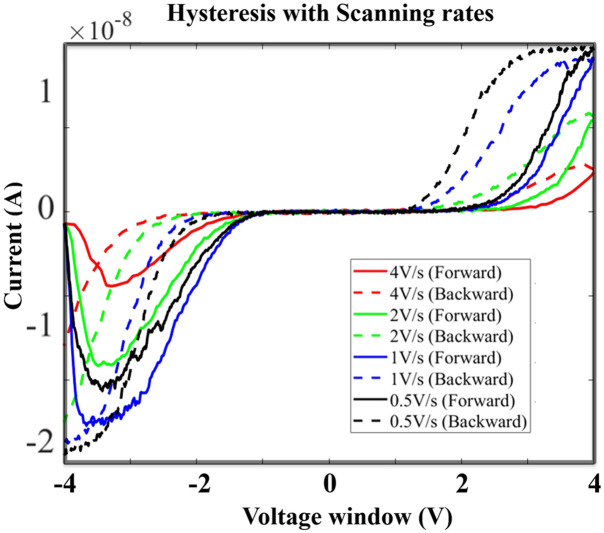
Memristive behaviour with scanning rates.

As originally predicted by the modeling proposed for 0D memristive devices^1^ the switching behaviour of memristors is expected going down with the sweeping-frequency of the applied voltage going up.^47^ Our memristive devices are subject to the same phenomenon as well. The source measure unit is used to demonstrate this property by supplying sweeps at different scanning rates. As shown in Fig. 5, memristive loops are obtained with scanning rates from 4 V s^−1^ down to 0.5 V s^−1^. However, it is evident that the extent of the hysteresis loop becomes more pronounced by decreasing the scanning rate. This observation corresponds to the result previously reported in the literature that shows smaller frequencies leading to more memristive properties.

Intuitively, we see that also using a simple expression in circuitry, *E* = *VIt*. It easily confirms that when the scanning rate decreases, which means a larger period *t*, the hysteresis loop moves towards the original point as shown in Fig. 5, meaning less voltage is required correspondingly. This simple energy-related consideration will guide us to the modeling and simulations shown later in section 4.

### 3.2. Schottky barrier tuning

**Fig. 6:**
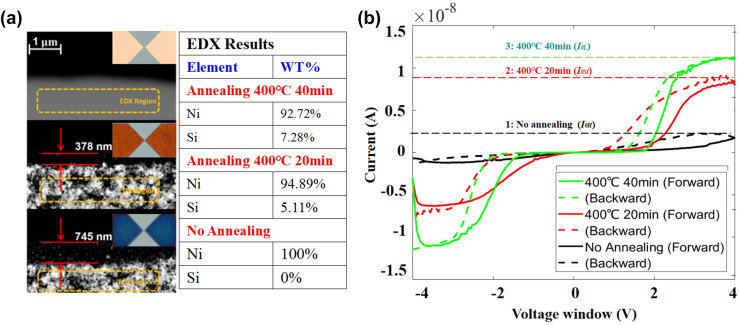
Schottky barrier tuning: (a) the Si–Ni contacts annealing observed in SEM images and (b) the memristive behaviour related to different annealing scenarios.

The SiNW-based device also incorporates two Schottky diodes. The impact of Schottky contacts is illustrated in Fig. 6 and demonstrates the effect of different annealing conditions on the Ni–Si contacts. The microscopic analysis shown in Fig. 6(a) reveals significant changes in the contact surface colour corresponding to different annealing extents. In the absence of annealing, the Ni surface appears rose coloured (picture (III) in Fig. 6(a)). When annealed at 400 °C for 20 minutes, the surface colour changes to orange (picture (II) in Fig. 6(a)). With further annealing, the colour further shifts to blue, indicating an increased intermixing of Ni and Si (picture (I) as directly observed in the SEM images in Fig. 6(a)).

Given the thin Ni layer, BSD is further employed from the top side to observe the atomic distribution at the edge of Ni deposition. Without annealing, the high SBH between Si and Ni results in an unfocused electron beam and a blurred image at 30k × magnification, as shown in the bottom image. Energy-dispersive X-ray (EDX) analysis in this case detects only Ni atoms. Annealed devices under conditions of 400 °C for 20 minutes and 400 °C for 40 minutes, as depicted in Fig. 6(a)I and II, exhibit the presence of NiSi grains on the surface. Atomic diffusion extends up to 375 nm from the Ni edge. EDX analysis reveals a composition of 5.11% Si and 94.89% Ni by mass. With prolonged annealing for another 20 minutes, the diffusion length increases to 825 nm, and the EDX analysis indicates a Si content of 7.28% and a Ni content of 92.72%.

The measured *I*–*V* curves are illustrated in Fig. 6(b), exhibiting a shape aligned to that in Fig. 5. The volatile memristive behaviour also shows a comparable trend when altering annealing conditions. As the device undergoes more extensive annealing, the saturation current also varies. The read-out saturation currents *I*_0H_ is 12.2 nA, *I*_0M_ is 7.8 nA, and *I*_0L_ is 2.3 nA under annealing conditions of 400 °C for 40 minutes, 400 °C for 20 minutes, and no annealing, respectively.

When Ni is in direct contact with Si, the SBH is determined by the difference in their work functions and the alignment of their Fermi levels. However, during annealing, Ni reacts with Si to form the compound nickel silicide. This reaction changes the electronic state density and chemical properties at the interface, resulting in a modified SBH. Furthermore, the compound phase has a distinct work function compared to pure Ni, which directly influences the barrier height concurrently; the experimental data in Fig. 6(b) demonstrate that the saturation current *I*_0_ increases with the annealing. Coherently with the eqn (1),

2 *I*_0_ ∝ exp (−*ϕ*_B_),

the saturation current *I*_0_ exhibits an inverse exponential relationship with the SBH (*ϕ*_B_). The observation of annealing aligns with theoretically decreasing SBH. Furthermore, the hysteresis loop width diminishes with higher saturation currents. Combined with the energy-related consideration *E* = *VIt*, a higher Schottky barrier results in a lower saturation current *I*_0_, and necessitates a higher switching voltage to compensate for the required energy for the increased potential (*ϕ*_B_) introduced by annealing treatment.

### 3.3. Intermediate oxide layer at the Schottky contacts

This memristive device features two basic components, the quasi-1D silicon channel and two Schottky contacts aside. Our previously described experiments have highlighted the influences of these two device-components on the memristive behaviour. It has been observed that robust volatile memristive behaviour emerges only in the presence of oxygen on the silicon channel. In this section, we further explore instead the role of an intermediate oxide layer between Ni and Si at the Schottky contact.

**Fig. 7:**
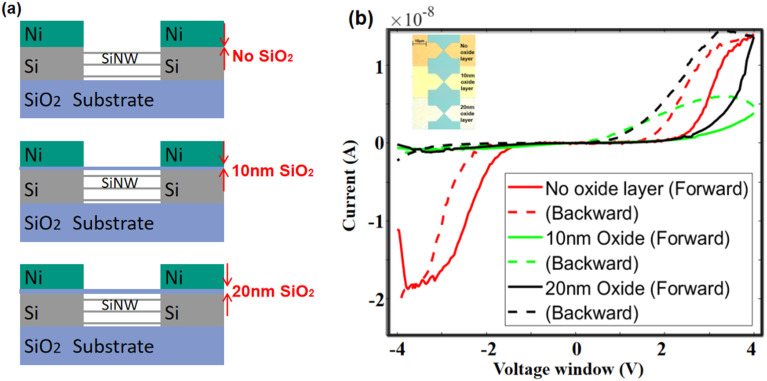
(a) Schematic of the intermediate oxide layer. (b) Memristive behaviour with different thicknesses of different intermediate oxide layers.

In this series of different fabrications, layers with various thicknesses of silicon oxide were sputtered onto the silicon-on-insulator chip before the deposition of Ni, in order to ensure the presence of an oxide layer between Ni and Si. Three scenarios are considered here: 0 nm, 10 nm, and 20 nm of oxide layer, as schematically sketched in Fig. 7(a). Under optical-microscopic examination, the standardized annealing process (400 °C for 20 or 40 minutes) results in Schottky contact surface colors of yellow or white, while no annealing results in orange as shown in Fig. 7(b). The *I*–*V* characteristics of these three different fabrications have been acquired after the oxygen plasma treatment (OPT) of the quasi-1D silicon channel and are presented as well in Fig. 7(b). In the absence of an oxide layer, the memristive behaviour, represented by the red curve, resembles that shown in Fig. 3(c). The green and black curves represent the cases of 10 nm and 20 nm oxide layers, respectively. The presence of the oxide layer alters the memristive behaviour, notably enlarging the hysteresis loop in the positive voltage region compared to the negative voltage region.

Fig. 7(b) shows that, at −4 V, the *I*–*V* curve for the 20 nm oxide layer (black curve) starts at a lower current compared to that of the 10 nm oxide layer (green curve). Interestingly, a thicker oxide layer corresponds to a higher maximum current. At the beginning of the backward sweep, the current continues to increase despite the voltage starting to decrease. This indicates a more complex underlying mechanism compared to that depicted in Fig. 3. The switching mechanism involving the intermediate oxide layer has been extensively discussed by Mehonic *et al.* in prior studies.^48^ In the forward sweep, initially at −4 V, the current is impeded by the insulating silicon oxide layer, resulting in significantly lower currents with respect to the current we measured at −3.9 nA. Then, the formation of conductive filaments within the oxide layer, driven by oxygen vacancies and ions, begins under the negative applied voltage.^49^ As the voltage continues to be applied, a substantial number of conductive bridges are formed, then leading to a scenario where the current continues to increase even as the applied voltage decreases during the backward sweep. The thicker oxide layer provides more oxygen vacancies, then more conductivity at the nanoampere scale.

## 4. Modelling and simulation

In order to deeply understand the experimental findings reported so far from a more comprehensive theoretical point of view, we have also developed a quite simple modelling of our device and obtained accordingly simulation outputs to demonstrate the main features of the device properties. The present section of the paper is dedicated to this theoretical aspect of our work.

### 4.1. The back-to-back diode structure

**Fig. 8:**
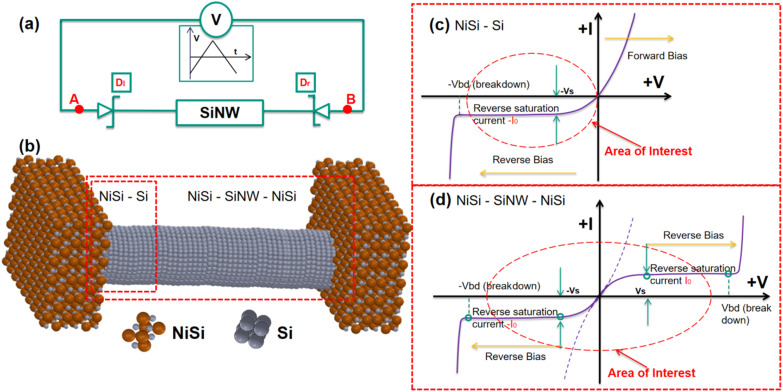
(a) Scheme of driving back-to-back Schottky diode structure with a triangular waveform signal. (b) Schematic of back-to-back Schottky diode structure formed across SiNW with a triangular signal. (c) *I*–*V* characteristics of a single diode. (d) *I*–*V* characteristics within a single voltage sweep through the back-to-back Schottky diode structure.

As depicted in Fig. 8(b), the back-to-back diode structure incorporates a conducting channel, and two Schottky diodes, denoted as Dl and Dr, subjected to a forward voltage sweep followed by a reverse sweep, as shown in Fig. 8(a). The voltage applied across the device can be distributed as the following:

3 *V*_AB_ = *V*_Dl_ + *V*_Si_ + *V*_Dr_.

In such a scheme, there is always at least one diode operating in a reverse-biased configuration. For instance, if the current flows from node A to B, diode Dl is forward biased while Dr is reverse biased. This is expressed as the following:

4 *R*_Dr_ ≫ *R*_Dl_, *R*_Dr_ ≫ *R*_Si_

which leads to,

5 *V*_AB_ ≈ *V*_Dr_

Fig. 8(c) presents a static *I*–*V* curve of a single diode. It comprises three distinct regions: the breakdown region, reverse saturation region, and thermionic emission region. The thermionic emission and the saturation current of the diode are both involved in the Shockley diode equation:^50,51^6
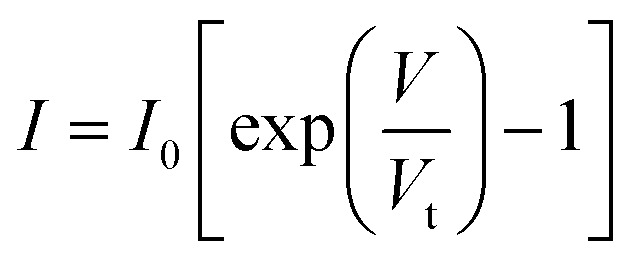


Here, the saturation current *I*_0_ is obtained using the eqn (1), and *V* represents the voltage applied across the diode. *V*_t_ = *ηk*_B_*T*/*q* is the thermal voltage, where *η* is the ideal factor usually chosen as 1^50,52^ in a regular Schottky diode. Eqn (5) suggests that the device operation is mainly governed by the reverse-biased diode. Thus, the area of interest labeled with a red frame in Fig. 8(c) is only the reverse biased region before breaking down. This region is defined as the following:

7 |*V*_AB_| < |*V*_bd_|.

Thus, the current going through the device in one direction is written as the following:8
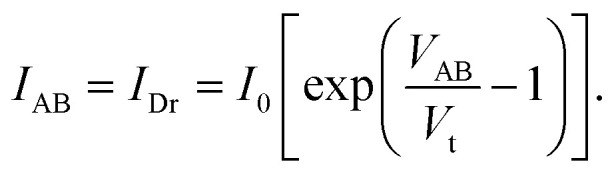


As discussed above, the electrical behaviour of the Schottky-barrier modulated SiNW can be described by the operation of a reverse-biased diode in both negative/positive cycles of the input signal. Fig. 8(d) shows an *I*–*V* characteristic model curve representing the integration of two back-to-back diodes. In the area of |*V*_AB_| < |*V*_S_|, the dashed line represents the dominance of carriers generated by the thermionic emission (gradual increase in current), while the solid current denotes the saturation due to the energy barrier (constant current). As a result, the conduction characteristic of the back-to-back diode structure can be split into five regions separated by the breakdown voltages and saturation voltages marked with green circles, where the area of interest is marked with a red frame as defined using eqn (7). Consequently, the current going through the device under voltage sweep can be described in eqn (9) within the “area of interest”:9
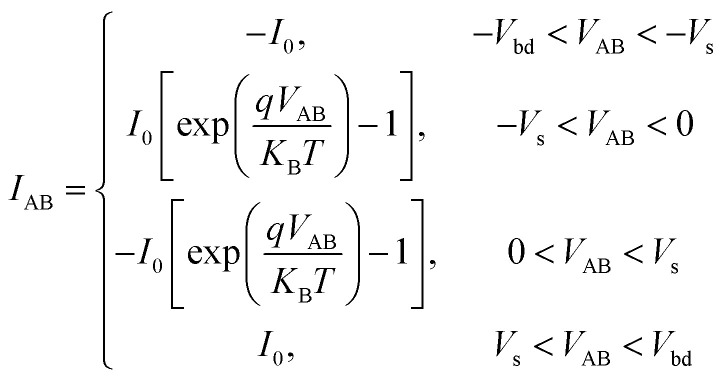


**Fig. 9:**
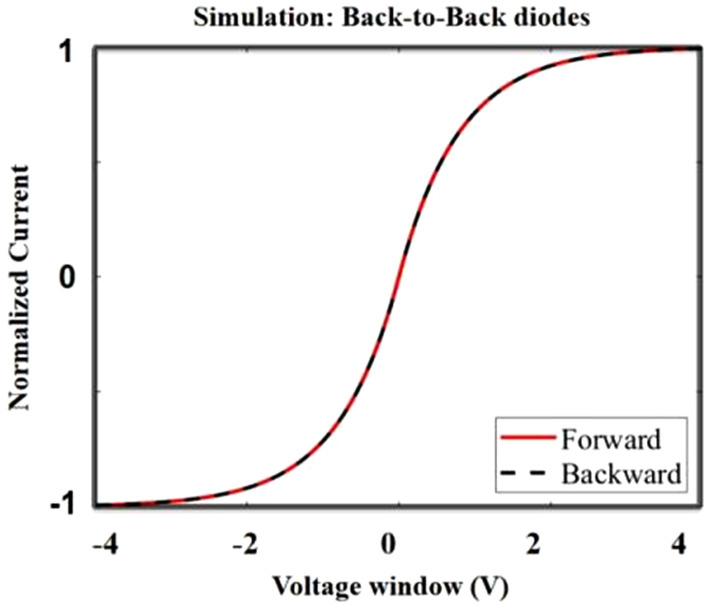
The simulated current of pure back-to-back diodes.

By using eqn (9), we conducted MATLAB mathematical simulations using an array as a window of input voltages to obtain an array of computed current values. The accordingly-obtained normalized *I*–*V* characteristics are depicted in Fig. 9. In addition, a brand-new fabricated device, as seen in section 2, manifests nearly superimposed curves during both forward and backward sweeps, as per the measured result in Fig. 2(a), which corroborates the model.

### 4.2. Volatile memristive behaviour

The volatile memristive behaviour is assumed to be energy-related as described in sections 3.1 and 3.2. In eqn (1), the (*qϕ*_B_) part is the energy term determined by the SBH (*ϕ*_B_). However, SBH is not constant in the actual diode. There is Schottky barrier lowering, also named as image force lowering, (*qϕ*_B_ − Δ*ϕ*_B_),^53,54^ which is governed by the drain and source bias, along with the energy consumption to overcome the energetic band separation from the conduction band, concluded in eqn (10):^55^

10 Δ*ϕ*_B_ ∝ (*ϕ*_B_ + *V*_R_ − *ϕ*_p_)^¼^

where *ϕ*_p_ is the potential difference between the valence band and the Fermi level of the p-type semiconductor, considered as a constant. A detailed introduction of the image force lowering effect can be found in ref 55. However, a precise relationship is still a subject of debate. The experimental plots delineated in a previous report^56^ provide empirical evidence supporting that the image force lowering is proportional to the square root of the electric field across the semiconductor depletion region, and the forward characteristic is less affected than the reverse characteristic. The prevailing consensus is that the reduction in barrier height exhibits a positive correlation with the applied voltage.^57^ Thus, an energy-related term is used to compensate for the impact of the input energy *E*_in_ on the *I*–*V* relationship using eqn (6), and is presented in eqn (11):11
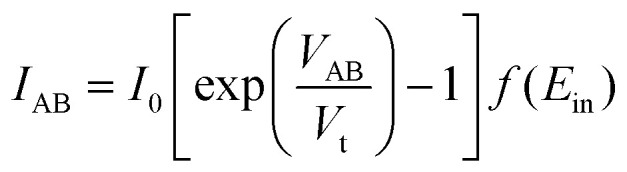
12
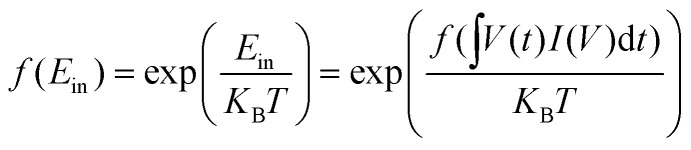


For an instantaneous moment, the instant input energy *δE*_in_ is defined as the following:

13 *δE*_in_ = *K*_s_ln (*VIδt*) + 1,

where *K*_s_ is the coefficient influenced by SBH. For an actual sweep, the integral is converted into a series, by

14 *E*_in_ = ∑*δE*^*n*^_in_.

Solving with an iterative way, the adjusted *I*–*V* relationship can be then described as the following:15
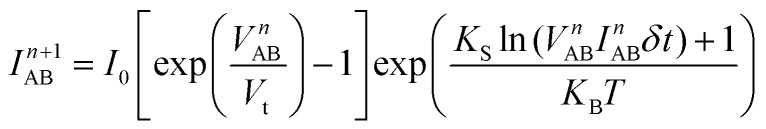


Finally, the mathematical model to simulate the memristive behaviour of back-to-back diode structure is written as eqn (16), combined with eqn (15).16
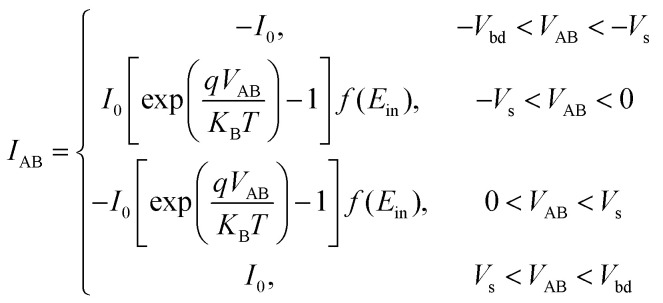


**Fig. 10:**
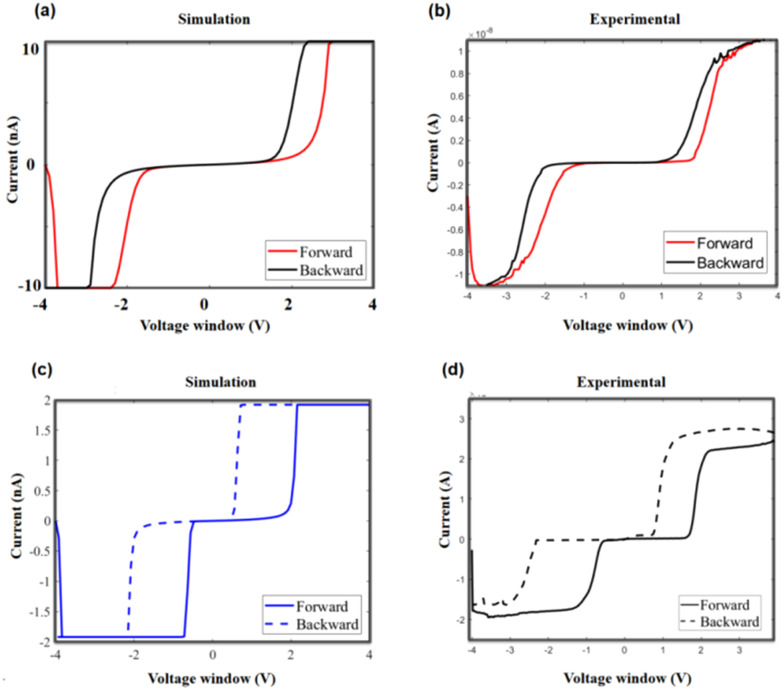
The comparison of simulated and experimental data. (a) The simulation results with a higher saturation current. (b) A representative measured *I*/*V* curve with an annealed device, showing a higher saturation current and small hysteresis. (c) The simulation results with a lower saturation current. (d) A representative measured *I*/*V* curve of the device without annealing, showing a lower saturation current and greater hysteresis.

By adjusting the parameters, the simulated results closely align with the experimental data, as demonstrated in Fig. 10. In Fig. 10(a), the simulated memristive behaviour is shown with *I*_0_ to 10 nA. The actual *I*–*V* curve, presented in Fig. 10(b), corresponds to a representative sample of an annealed device with a saturation current of approximately 12 nA. When *I*_0_ is reduced to 2 nA as shown in Fig. 10(c), the memristive behaviour exhibits a more pronounced hysteresis. This aligns with the behaviour of an unannealed device, which has a higher SBH. Correspondingly, as seen in Fig. 10(d), an unannealed device with a SBH shows a saturation current of around 2 nA, matching the simulated behaviour. This comparison confirms that the simulation results accurately replicate the actual device characteristics under different conditions, demonstrating a strong alignment between the model and experimental observations. For more details on simulation, see the ESI, in Fig. S2 and S3† for the frequency-dependent behaviour and Schottky barrier influence, respectively.

## 5. Discussion

The proposed model is simplified, focusing primarily on the energy-related term under ideal conditions and neglecting factors such as interface defects and parasitic effects. Despite these simplifications, the comparison between simulated and experimental data shows no significant discrepancies, as the simulation successfully incorporates the key features of volatile memristive behaviour.

**Fig. 11:**
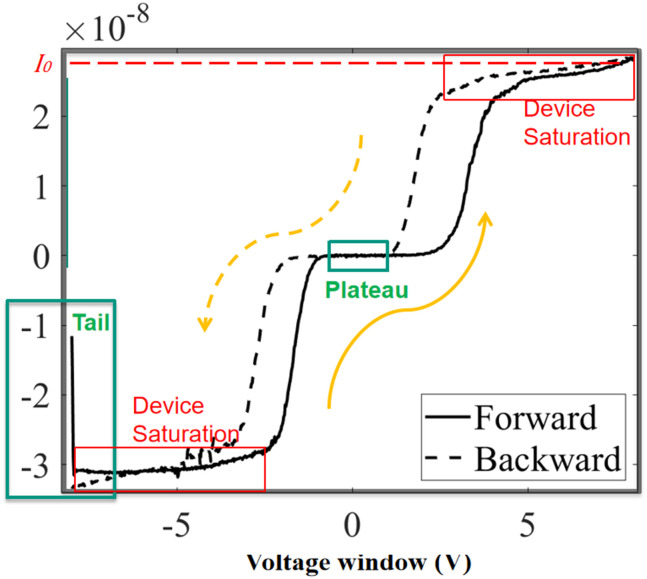
The key features of a complete non-crossing anti-clockwise hysteresis loop with an input voltage window (8 V).

To better understand this type of volatile memristive behaviour, an 8 V voltage window is used to highlight the key features, as shown in Fig. 11:

### 5.1. Tail effect

When the sweep begins, a “tail” appears, representing a sudden conductance change, consistent with the behaviour shown in Fig. 4.

### 5.2. Central plateau

In the central region, the current remains nearly zero, forming a plateau. This can be explained by the energy-compensation assumption, where the device requires sufficient energy input to overcome the high-resistive state.

### 5.3. Saturation current

The current saturates at a specific value (*I*_0_) due to the rectification effect of the Schottky contacts. Even at higher input voltages, the current remains almost constant around *I*_0_.

### 5.4. Non-crossing, anti-clockwise hysteresis

The *I*–*V* curve exhibits non-crossing, anti-clockwise hysteresis.

The simulation using eqn (16) successfully reproduces these four key features, aligning well with the experimental observations.

Notably, for this single device shown in Fig. 11, the saturation current in the negative region is higher than that in the positive region, attributed to fabrication errors at the Schottky contacts. Consequently, the hysteresis in the negative region is noticeably thinner than that in the positive region. This behaviour is consistent with the results shown in Fig. 6, where a higher SBH leads to more pronounced memristive hysteresis.

This observation underscores that, although unpredictable errors may occur during actual fabrication, the overall results remain highly consistent with the fundamental principles derived in this paper. This demonstrates that, even under complex fabrication conditions, the proposed model and principles are highly applicable and reliable, offering both theoretical guidance and experimental validation for the further optimization and design of similar devices.

## 6. Conclusion

This report investigates the role of the oxide in quasi-1D memristive devices made with silicon nanowires, typically proposed over the last decade of published experiments for memristive biosensors. These kinds of memristive devices comprise two Schottky contacts and stacks of SiNWs. Thanks to the oxidation of SiNWs, the devices demonstrate robust volatile memristive behaviour along with the expected frequency-dependent properties. Additionally, we have demonstrated that tuning the SBH of the metal contacts significantly affects the device's characteristics. The higher SBH results in a greater extent of hysteresis. Furthermore, the influence of the intermediate oxide layer placed in between the Schottky contacts has been also investigated and discussed. This oxide layer acts to amplify the hysteresis. Finally, a model based on thermal emission has been proposed grounded in the energy-related assumptions. This model is actually in very good alignment with the observed experimental measurements.

The several pieces of evidence investigated in this report also play a key role in designing and optimizing memristive biosensors. Therefore, this research ultimately paves the way for a better development of new categories of volatile memristive devices and guides further more precise designs in memristive biosensing.

## Author contributions

J. Chen: writing – review & editing, writing – original draft, methodology, experiments, simulations, investigations, and formal analysis. S. Carrara: writing – review & editing, conceptualization supervision, validation, project administration, funding acquisition, and resources. K. Bhardwaj: writing – review & editing, methodology, and simulations.

## Data availability

Data for this article are available at Github at https://github.com/JunruiChen666/Memristive-behavior.

## Conflicts of interest

There are no conflicts to declare.

## Supplementary Material

NR-017-D5NR00104H-s001
