# Supplementary material for: Role of the oxide in memristive quasi-1D silicon nanowires
Source: Nanoscale. 2025 Feb 19;17(14):8660–71. doi: 10.1039/d5nr00104h (PMC11894604; doi:10.1039/d5nr00104h)
Supplement: NR-017-D5NR00104H-s001 [file NR-017-D5NR00104H-s001.pdf]

Supplementary Information (SI) for Nanoscale.
This journal is © The Royal Society of Chemistry 2025

Title: Role of the Oxide in Memristive Quasi-1D Silicon Nanowire

Authors' Names: Junrui Chen[a]*, Kapil Bhardwaj[2] and Sandro Carrara[1]

[1]Bio/CMOS Interfaces Laboratory (BCI), École Polytechnique Fédérale de Lausanne, 2000 Neuchâtel, Switzerland

[2]Department of Electronics and Communication Engineering, National Institute of Technology, Jamshedpur, Jharkhand, 831014, India

Email Address:

junrui.chen@epfl.ch*

Supplementary Information

S1a:

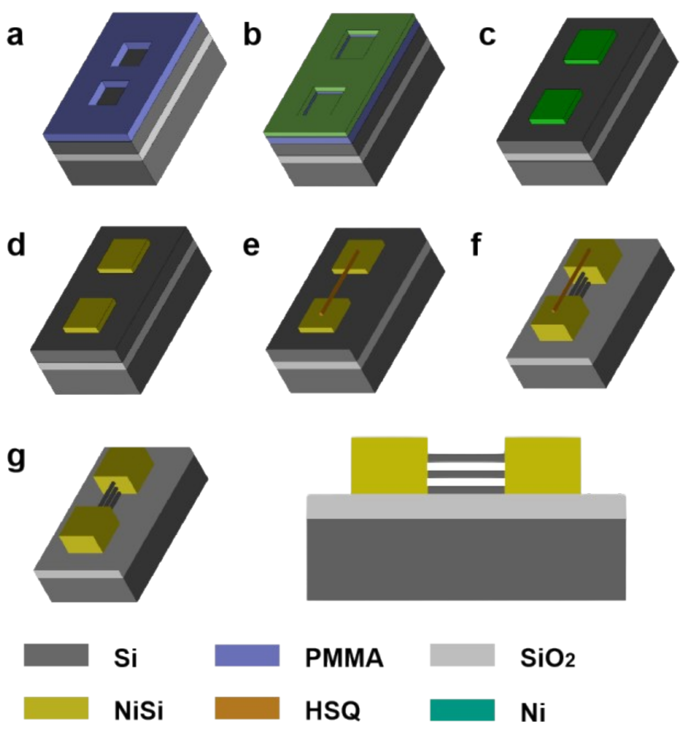

S1b:

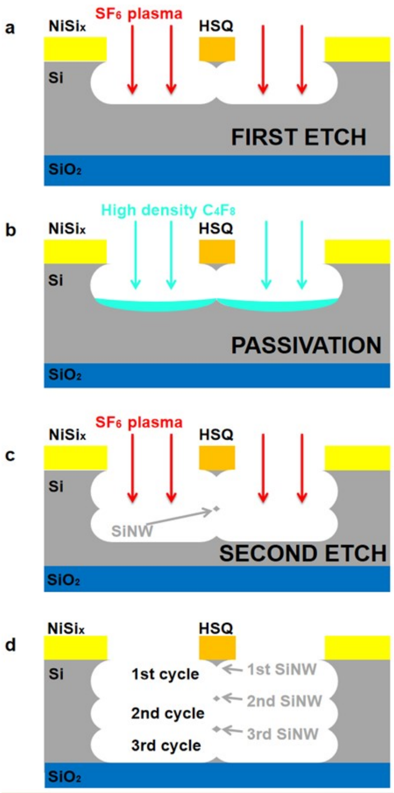

Figure S1: (a) Schematics of SiNW fabrication process. a: Ebeam-resist spin-coating, b: Nickel evaporation, c: Resist removal, d: Annealing for silicidation, e: HSQ definition for nanowires, f: Bosch process for Si etching, g: HSQ removal. (b) The silicon nanowires form with the alternating etching and passivation gas. a: The over-etching under the HSQ resist in the first cycle with SF_6 plasma. b: The passivation with high density C_4F_8. c: Silicon etching in the second cycle (Passivation is not showing). d: The stacked silicon nanowires after repeated etching cycles.

As presented in the Figure S1a, a standard fabrication process for SiNWs begins with a top-down, CMOS-compatible technique on a p-type silicon-on-insulator (SOI) substrate with a resistivity range 14-22 Ωcm. The substrate consists of a thin silicon layer and a buried oxide layer, supported by a thicker silicon wafer. To define the pattern for the Ni Pads, a layer of polymethyl methacrylate (PMMA) (a) is patterned using Electron Beam Lithography (EBL) in step (b). Subsequently, a 50 nm thick layer of Nickel (Ni) is evaporated onto the surface.

In step (c), the excess PMMA layer is removed with acetone, followed by ultrasonic rinsing for 20 minutes. Moving on to step (d), the substrates, now carrying the patterned Ni layer, undergo a crucial annealing step in an inert N2 ambient under varying annealing conditions. The default annealing condition is at 400 °C for 20 minutes without a specific definition. This annealing process initiates a reaction between Ni and silicon from the substrate, forming Schottky contacts.

For the subsequent step (e), another Electron Beam Lithography (EBL) process is employed, this time using a 100 nm thick layer of Hydrogen Silsesquioxane (HSQ) as a negative-tone resist. This resistance is essential for defining the dimensions and layout of the silicon nanowires. Moving forward, the substrate undergoes Deep Reactive Ion Etching (DRIE), also known as the "Bosch Process," to create the silicon nanowires in step (f). Lastly, in step (g), the HSQ resist is stripped in a solution comprising 1% HF for 1 minute. The application of 1% HF serves a dual purpose: it strips the HSQ resist and potentially eliminates oxide layers from the SiNW surface.

As presented in Figure S1b, this process involves alternating etching and passivation steps using SF_6 (etching gas) and C_4F_8 (passivation gas) in a cyclic manner. In the first cycle, by tuning the etching time, SF_6 gas over-etches the silicon (step a), leaving a smaller arc will appear on the wall and in the center. Then the C_4F_8 gas passivates the side wall and the center (step b). When the bottom silicon is etched in the 2nd etching circle (step c), a silicon nanowire is formed in the center. During the repeated etching and protection, the nanowires are left behind. As a result, stacked silicon nanowires are formed, leaving them suspended between the NiSi contacts (d).

S2:

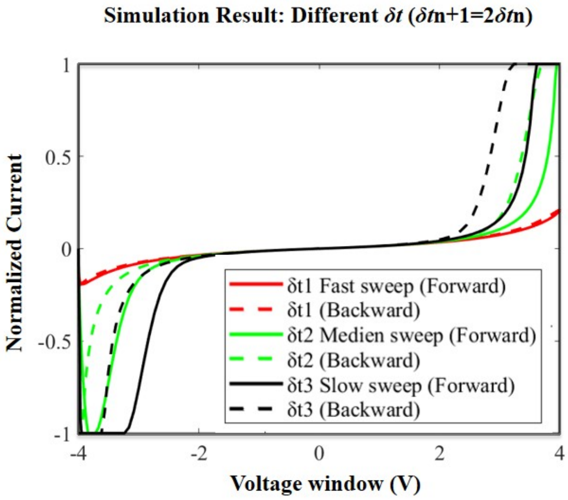

S2: Plots generated using Equation 15 with varying operating scanning rates

To simulate the frequency-dependent behavior of the model in Equation 15, the for- and backward sweeps voltages are defined with sinus signals. This model clearly demonstrates a decreasing hysteresis with increasing frequency, similar to figure 5.

```
dt=[0.01, 0.02, 0.04]; % Time span
step =100; % sweep numbers
T= 2*step * dt; % Periode, forward and backward
t=[0:dt:T]; % Time array
V=Vin*sin((1/dt)*(pi/step)*t) % Sinus input, sampling rate >> frequency
% Sinus input, frequency = 1 / T
Vfp = V(1:step/2);  %Forward positive sweep
Vbp = V(step/2+1:step); %Back positive sweep
Vbn=V(step+1:1.5*step); %Back negative sweep
Vfn = V(1.5*step+1:end); %Forward negative sweep
```

S3.

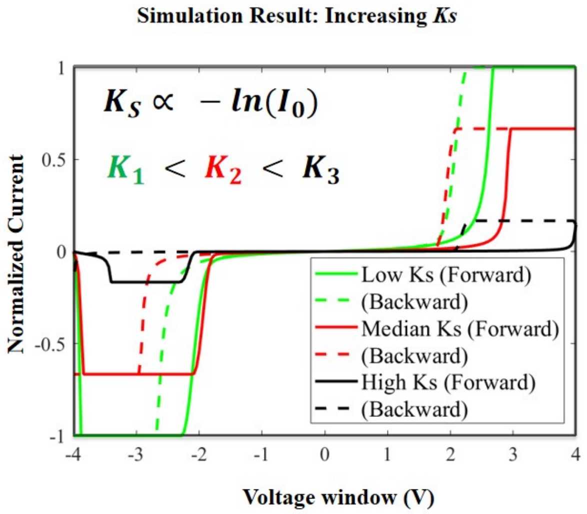

S3: Simulations result for Equation 15 with different Ks values

Ks is defined as a Schottky barrier height related coefficient. Combining Equation 2, it can be described as:

$K_S \propto -ln(I_0)$.

As a result, the simulated results in Figure S3 present similar result as figure 6b. A higher Schottky barrier height leads to smaller saturation current $I0$ and greater hysteresis. The main difference is that the simulated model pretends to be a digital switching, while the actual data show a more analog switching. The definitions of digital & analog switching can be found in [1].

Reference:

[1]: R. Wang, J.-Q. Yang, J.-Y. Mao, Z.-P. Wang, S. Wu, M. Zhou, T. Chen, Y. Zhou, S.-T. Han, Recent advances of volatile memristors: Devices, mechanisms, and applications, Advanced Intelligent Systems 2 (2020) 2000055.
